# Polysaccharides, Next Potential Agent for the Treatment of Epilepsy?

**DOI:** 10.3389/fphar.2022.790136

**Published:** 2022-03-18

**Authors:** Xuemin Xie, Youliang Wu, Haitao Xie, Haiyan Wang, Xiaojing Zhang, Jiabin Yu, Shaofang Zhu, Jing Zhao, Lisen Sui, Shaoping Li

**Affiliations:** ^1^ Department of Epilepsy Center, The Second Affiliated Hospital of Guangzhou University of Chinese Medicine (Guangdong Provincial Hospital of Chinese Medicine), Guangzhou, China; ^2^ Joint Laboratory of Chinese Herbal Glycoengineering and Testing Technology, State Key Laboratory of Quality Research in Chinese Medicine, Institute of Chinese Medical Sciences, University of Macau, Macao, China

**Keywords:** polysaccharides, epilepsy, traditional Chinese medicines, gut microbiome, treatment

## Abstract

Epilepsy is a chronic neurological disorder. Current pharmacological therapies for epilepsy have limited efficacy that result in refractory epilepsy (RE). Owing to the limitations of conventional therapies, it is needed to develop new anti-epileptic drugs. The beneficial effects of polysaccharides from Chinese medicines, such as *Lycium barbarum* polysaccharides (COP) and *Ganoderma lucidum* polysaccharides (GLP), for treatment of epilepsy include regulation of inflammatory factors, neurotransmitters, ion channels, and antioxidant reactions. Especially, polysaccharides could be digested by intestinal microbial flora, referred as “intestinal brain organ” or “adult’s second brain”, may be the target for treatment of epilepsy. Actually, polysaccharides can effectively improve the type and quantity of intestinal flora such as bifidobacteria and lactic acid bacteria and achieve the purpose of treating epilepsy. Therefore, polysaccharides are hypothesized and discussed as potential agent for treatment of epilepsy.

## 1 Introduction

Epilepsy is a chronic neurological disease that affects more than 50 million people worldwide and accounts for 0.6% of the global economic disease burden (WHO, 2016). Although WHO estimates that the seizures can be controlled by appropriate medications in 70% of epilepsy patients, only less than half of them have access to antiepileptic drugs in developing countries. In addition, there is still an estimated of 15 million patients have refractory epilepsy (RE) due to the pool response to existing anti-epileptic drugs (Lum et al., 2020). Therefore, RE has become a hot research topic of neurological treatment. In the past few decades, more than 20 kinds of anti-epileptic drugs have been developed while the incidence of RE has not been significantly reduced. The commonly used clinical antiepileptic drugs include sodium valproate (VPA), carbamazepine, phenobarbital, phenytoin sodium, ethylamine, oxcarbazepine, lamotrigine, topiramate, levetiracetam, lacoamide and so on. Each drug has its own unique physiological activity. For example, VPA is a broad-spectrum antiepileptic agent that can be used as either monotherapy or adjunctive therapy for generalized epilepsy. Common adverse reactions of VPA include gastrointestinal reaction, liver injury, tremor, increased sleep, and long-term application may include weight gain, hair loss, menstrual disorders, polycystic ovary syndrome, etc. Compared with carbamazepine, oxcarbazepine is characterized by weak liver enzyme induction, high bioavailability, better efficacy and safety. oxcarbazepine is mainly used for partial epilepsy in children. Common adverse reactions of oxcarbazepine include nausea, dizziness and diplopia. It is important to note that these drugs require long-term or even lifelong use, which makes patients more prone to hematopoietic damage, Stevens-Johnson syndrome, severe liver dysfunction, and aggravated cognitive impairment. Considering the limitation of anti-epileptics, it is need to develop new drugs with lower side effects and higher efficacy (Mehla et al., 2010). In last years, more and more studies have shown that chemicals in traditional Chinese medicines (TCMs), such as triglycerides and saponins, could be used as therapeutic agents for epilepsy by regulating inflammatory factors, neurotransmitters, ion channels and antioxidant responses (Zhang et al., 2019), which has unique advantages such as low side effects and reduced complications (Yuan et al., 2019). Polysaccharides might also be a potential agent for treatment of epilepsy. The therapeutic efficacy may be mainly derived from their prebiotic effect on gut microbiota.

## 2 Gut Microbiota and Epilepsy

Gut microbiota, which is called “intestinal brain organ” and “adult’s second brain”, is related to metabolic diseases, autoimmune diseases, and nervous system diseases (Pellegrini et al., 2018). Recent studies indicate that gut dysfunction/dysbiosis is presumably involved in the pathogenesis of and susceptibility to epilepsy. In addition, the reconstruction of the intestinal microbiome through, for example, faecal microbiota transplantation, probiotic intervention, and a ketogenic diet, has exhibited beneficial effects on drug-resistant epilepsy (Yue et al., 2021). Indeed, a few recent studies have highlighted differences in fecal microbiota profiles from selected epileptic individuals as compared to healthy controls (Lum et al., 2020). In addition, a 22-year-old Crohn’s disease patient with a 17-year history of seizures underwent a fecal microbiota transplant to treat Crohn’s disease (He et al., 2017). During the 20-month follow-up, the patient had no seizures despite discontinuation of the antiepileptic treatment with sodium valproate. Another study showed that probiotic treatment reduced the frequency of seizures by more than 50% in 28.9% of patients with drug-resistant to epilepsy (Gómez-Eguílaz et al., 2018). It was found that intestinal dysbiosis is associated with chronic stress-induced epilepsy in rats and members of the intestinal microbiota influence the anti-seizure effect of the ketogenic diet in mice. Recent studies in human cohorts suggest a dysbiosis in children with epilepsy. It may be possible that dysbiosis is more relevant in certain subtypes of epilepsy though larger studies with age-matched controls are needed to confirm (Dahlin and Prast-Nielsen, 2019). Braakman and Ingen described 6 cases of drug-resistant epilepsy, of which 5 had no seizures and 1 had a reduction in seizure frequency by more than 90% during antibiotic treatment. This effect disappeared within 2 weeks of stopping treatment, presumably due to the recovery of certain gut microbes (Braakman and Van Ingen, 2018) though other potential mechanisms might not be excluded (Sander and Perucca, 2003). Peng et al. (2018) found that the abundance of rare intestinal flora in patients with drug-resistant epilepsy increased abnormally, and the number of beneficial bacteria such as bifidobacteria and lactobacilli decreased. Olson et al. (2018) found that the ketogenic diet changes intestinal flora, which is a necessary part of the ketogenic diet, to exert its anti-epileptic effect. This information is linked to that a decreased amount of long-chain (such as arachidic, and oleic acid) and medium-chain fatty acids (sebacic acid and isocaproic acid) as well as bile acid was observed in patients with inflammatory bowel disease (Weng et al., 2019). Indeed, sebacic acid (SA) is a component of ketogenic diet and administered in pure form to inhibit the P-glycoprotein function and expression in an experimental model of refractory epilepsy (Enrique et al., 2021). Those above studies have indicated that regulating gut microbiota can achieve therapeutic effect of epilepsy. There are multiple interactions between gut microbiota and central nervous system (CNS). Gut microbiota affects the development and homeostasis of CNS through immune, circulatory and neural pathways, while CNS induces gut microbiota through stress and endocrine responses (Dinan and Cryan, 2017; Tremlett et al., 2017). The term “brain gut axis” is used to describe these two-way interactions (Bauer et al., 2016). Bagheri et al. found that there was a significant imbalance of intestinal flora in experimentally induced epileptic rats, and there was a certain proportion between the dominant flora in intestinal flora and seizures (Bagheri et al., 2019). The concentration of the inhibitory neurotransmitter gamma-aminobutyric acid (GABA) increased and the severity of epileptic seizures was significantly reduced in rats treated with probiotic supplements. This may be related to the fact that selective probiotics modulate the expression of specific GABA receptor subunits in brain regions (Liang et al., 2017). Studies have found that intestinal microflora α-diversity significantly increased in patients with refractory epilepsy, and the lower level of *bifidobacteria* and *lactobacillus*, the more frequent the seizures. Recently, some scholars have observed that intestinal flora can regulate the function of CNS in multiple ways and can affected epileptic seizures. In addition, intestinal microflora disorder may be caused by regulating immune and inflammatory responses, changing nutrient metabolism, activating and improving microglia and astrocyte functions, changing vagal nerve activity, and reducing neuroactive substances in limbic system such as hippocampus [such as brain-derived neurotrophin (neurotrophic factor)] increased the risk and susceptibility of epilepsy (Vuong et al., 2017). Therefore, it is believed that the intestinal flora may be a target for RE treatment (De Caro et al., 2019; He et al., 2021). The gut-brain bidirectional axis and the underlying mechanism of KD-based therapy targeting gut microbiome in *in vivo* animal models and clinical studies in neurological diseases have been reviewed (Rawat et al., 2020). Briefly, the intestinal microbiota can affect the balance of excitement/inhibition through neurotransmitters (mainly GABA, glutamate and 5-HT) or their precursors (such as tryptophan) (O'Mahony et al., 2015; Mittal et al., 2017), thereby affecting the occurrence and maintenance of epileptic seizures and the occurrence of epilepsy. Immune system-mediated pro-inflammatory effects (for example, the release of cytokines and chemokines) also increase the level of LPS due to the passage of the intestinal barrier. Increased permeability (Belkaid and Hand, 2014; Blander et al., 2017) and production of short-chain fatty acids (especially butyric acid, propionic acid and acetic acid) has an anti-inflammatory effect (Stilling et al., 2016; van de Wouw et al., 2018). In addition, neural (such as vagus nerve afferent, enteric nervous system) and neuroendocrine (such as hypothalamus-pituitary-adrenal axis) networks (Sudo et al., 2004; Cheung et al., 2019), as well as the endocannabinoid system (Rousseaux et al., 2007) and brain-derived neurotrophic factor level, may be affected by gut microbiota, and therefore further effect on the seizure mechanism. Many highly modifiable gut microbiota–brain axis pathways may be related to epilepsy.

## 3 Effects of Polysaccharides on Epilepsy Through Gut Microbiota

Polysaccharides are biological macromolecules formed by the polymerization of more than 10 monosaccharides through glycosidic bonds. They are widely found in animals, plants and microorganisms. Indeed, TCMs are usually administered as decoction which contains larger proportion of polysaccharides. In recent years, with the development of “glycobiology”, studies have found that polysaccharides not only participate in various physiological activities, but also have a wide range of biological effects (Chen et al., 2016). Since lack of polysaccharides hydrolase, most polysaccharides cannot be directly digested and absorbed by human body. Intestinal flora play a mediating role in the process of interaction between polysaccharides and human body. Most of the beneficial health effects of polysaccharides have been associated with its reversal impacts on gut microbiota dysbiosis (Wang et al., 2021b). Mental illnesses, such as depression, Parkinson’s disease, Alzheimer’s disease and autism have been linked to gut microbiota. Actually, *Flammulina velutipes* polysaccharides contributed to significant improvements in mice learning and memory behavior through its gut microbiota regulation (Ma et al., 2021). Polysaccharides may also mainly contribute to their treatment of epilepsy through gut microbiota. The potential effects of polysaccharides from edible mushroom *Grifola frondosa* (GFP) on gut microbiota dysbiosis were investigated (Li et al., 2019). Metagenomic analysis revealed that GFP supplementation (400 mg/kg/day) resulted in significant structure changes on gut microbiota in high-fat diet (HFD)-fed rats, in particular modulating the relative abundance of functionally relevant microbial phylotypes compared with the HFD group. SP2-1, one homogeneous polysaccharide isolated from *Scutellaria baicalensis* Georgi can repair the intestinal barrier through up-regulated expressions of ZO-1, Occludin and Claudin-5. Furthermore, as compared with model group, the abundance of Firmicutes, Bifidobacterium, *Lactobacillus*, and Roseburia were significantly increased and the levels of *Bacteroides*, Proteobacteria and *Staphylococcus* were significantly inhibited with SP2-1 treatment. The modulatory effects of jujube (*Ziziphus jujuba* Mill.) polysaccharides (ZJP) on intestinal microbiota were investigated and the gut flora structure was then analyzed using high-throughput sequencing. After ZJP treatment, there was a significant decrease in Firmicutes/Bacteroidetes, which suggested that ZJP showed prebiotic-like activities by positively modulating intestinal microbiota (Ji et al., 2020). *Lycium barbarum* polysaccharides (LBPS) treatment also could modulate the composition of the gut microbiota, increasing the relative abundances of Bacteroidaceae, Lactobacillaceae, Prevotellaceae and Verrucomicrobiaceae, which were positively associated with immune traits. The present results indicated that LBPS might regulate the immune response depending on the modulation of the gut microbiota, suggesting that LBPS could be developed as special ingredients for immunoregulation in association with the modulation of the gut microbiota (Ding et al., 2019).

A few studies provided evidence that intestinal inflammation was also a contributing factor to epileptic events for susceptible patients and a possible reason for the reduced efficacy of antiepileptic drugs, which made intestinal inflammation a promising antiepileptic drug target (Yue et al., 2021). Many Chinese herbal polysaccharides have immune regulation functions such as protecting the body’s immune organs, activating immune cells, activating the complement system, and releasing cytokines (Hong et al., 2019; Zeng et al., 2019; Xi et al., 2020; Wang et al., 2021a), these are beneficial to the treatment of epilepsy. In the early stage of epilepsy, excessive reactive oxygen species (ROS) free radical is produced in the body, causing inflammation. Polysaccharides are beneficial for the treatment of epilepsy through antiinflammation, regulating excitatory neurotransmitters and receptors, sodium/potassium ion channels and antioxidant activities (Yuan et al., 2019). *Dendrobium officinale* polysaccharides have anti-inflammatory, antioxidative and immunity-enhancement effects, which attribute to the treatment of epilepsy due to their strong anti-inflammatory and antioxidative effects (Zhang et al., 2019). *Cornus officinalis* fruit polysaccharides reduce the activation of ROS and Mitogen-activated protein kinaseMAPK cascade pathways in hippocampus after epilepsy, the change of mitochondrial membrane potential, the leakage of cytochrome C, and the activation of cleaved caspase-3, thereby reducing neuronal apoptosis and having neuroprotective effects on epilepsy (Sun et al., 2018). *Glycyrrhiza uralensis* polysaccharides (GUP) may inhibit the oxidative stress and inflammation in epileptic rats ignited by pentylenetetrazol by down-regulating the expression of hippocampal P2X7 receptor and NF-κB protein, and reduce neuropathological damage (Xiao et al., 2021). Additionally, after the intervention of LBPS in epilepsy model rats, the number of BrdU-positive cells in the granular layer of the hippocampus dentate gyrus, the expression of MAP-2 and NeuN-positive neurons were improved to a certain extent, and it has a good neuroprotective effect (Feng et al., 2017). LBPS also can improve the learning and memory ability of epileptic rats, and its mechanism may be related to the protection of hippocampal neurons by enhancing the anti-oxidative stress effect (Chen et al., 2020). GLP may increase the expression of GLAST, GLT-1 and EAAC1 to reduce neuronal excitability and reduce or inhibit epileptic seizures (Zhu et al., 2015). It may reduce the influx of calcium ions in nerve cells, thereby indirectly inhibiting the activation of NF-κB induced by pentylenetetrazol, reducing the excitability of nerve cells, and achieving anti-epileptic effects (Zhang et al., 2010). Table 1 summarized some TCMs polysaccharides and their effects on cell culture or animal model, and the mechanism.

**TABLE 1:** Components from TCMs and their effects and cell culture or animal models.

TCMs component	Model	Mechanisms	References
GLP	NF-KB in hippocampal nerve cells of epileptic rats	Reduce the influx of calcium ions in nerve cells,so as to indirectly inhibit the activity of nerve cells	Zhang J et al. (2018)
	excitatory amino acid transporter in brain of epileptic rat	Glutamate transporter	Zhu K et al. (2015)
		GLAS↑GLT1↑EAAC1↑	
ASP	lipopolysaccharide-evoked inflammatory injury in neuronal cell line HT22	interdicted NF-jB and JAK2/STAT3 pathways via enhancing miR-10a	Zhou Y et al. (2019)
LBP	epileptic rats induced by lithium chloride-pilocarpine	Number of BrdU positive granular cells ↑	Feng et al. (2017)
		Map-2 positive neuron perimeter ↑	
	hippocampal neural stem cell	Differentiation rate of NeuN positive neurons↑	
	epileptic rats induced by lithium chloride-pilocarpine	The escape latency↓	Chen et al. (2020)
	hippocampus	The percentage of plateau quadrant path and plateau quadrant residence time↑	
COP	epileptic rats induced by lithium chloride-pilocarpine	SOD↓MDA↑mitochondrial ROS↑	Sun X et al. (2018)
		The mitochondrial membrane potential↓cytochrome C and activation of cleaved-caspase-3↓	
		MAPK phosphorylation↑	
		Morphological abnormalities with neurons in CA1 area of hippocampal were alleviated	
GUP	Epileptic rat model was established by pentylenetetrazol kindling	SOD↑MDA↓IL-18↓TNF-α↓	Xiao J et al. (2021)
DOP	Effects of Dendrobium Officinale Polysaccharides on Brain Inflammation of Epileptic Rats	IL-1β↓TNF-α↓ MAPK↓	Zhang L et al. (2019)
		P-mcp-1↑	
DOP	Effects of Dendrobium Officinale Polysaccharides on Brain Inflammation of Epileptic Rats	IL-1β↓TNF-α↓ MAPK↓	Zhang L et al. (2019)
		P-mcp-1↑	
GBPw	Middle cerebral artery occlusion rats	MDA↓ TNF-α↓ and IL-1β↓ SOD↑ MPO ↑ IL-10↑	Yang Y et al. (2013)
DOPS	Animalmodels of learning andmemory disabilitie	ctivateNrf2/HO-1 pathway to reduce oxidative stress and neuro-inflammation	Liang J et al. (2019)
LJPB2	Focal ischemia/reperfusion (I/R) injuried rat brain	SOD↑ GSH-Px↑	Su D et al. (2017)
GPP1	PC12 cells	Prevent Aβ(25–35)-induced oxidative stress, excessive [Ca2+]i influx, loss of mitochondrial membrane potential (ψm) and elevation of Bax/Bcl-2 protein expression and cleaved caspase-3, or by some combination of these effects	Jia D et al. (2015)
APS	APP/PS1 mice	activates Nrf2 pathway to regulate oxidative stress, improve apoptotic level and cognitive ability and reduce the accumulation of Ab	Qin X et al. (2020)

GLP, *Ganoderma lucidum* polysaccharides; ASP, *Angelica sinensis* polysaccharides; LBP, *Lycium barbarum* polysaccharides; COP, *Cornus officinalis* fruit polysaccharides; GUP, *Glycyrrhiza uralensis* polysaccharides; DOP, *Dendrobium officinale* polysaccharide; GBPw, an araban type polysaccharide was purified from the leaves of *Ginkgo biloba*; DOPS, *Dendrobium officinale* polysaccharides; LJPB2, a polysaccharide from Lonicera japonica flowers; GPP1, a purified polysaccharide from *Gynostemma pentaphyllum*; APS, *Astragalu*s polysaccharide.

**FIGURE 1:**
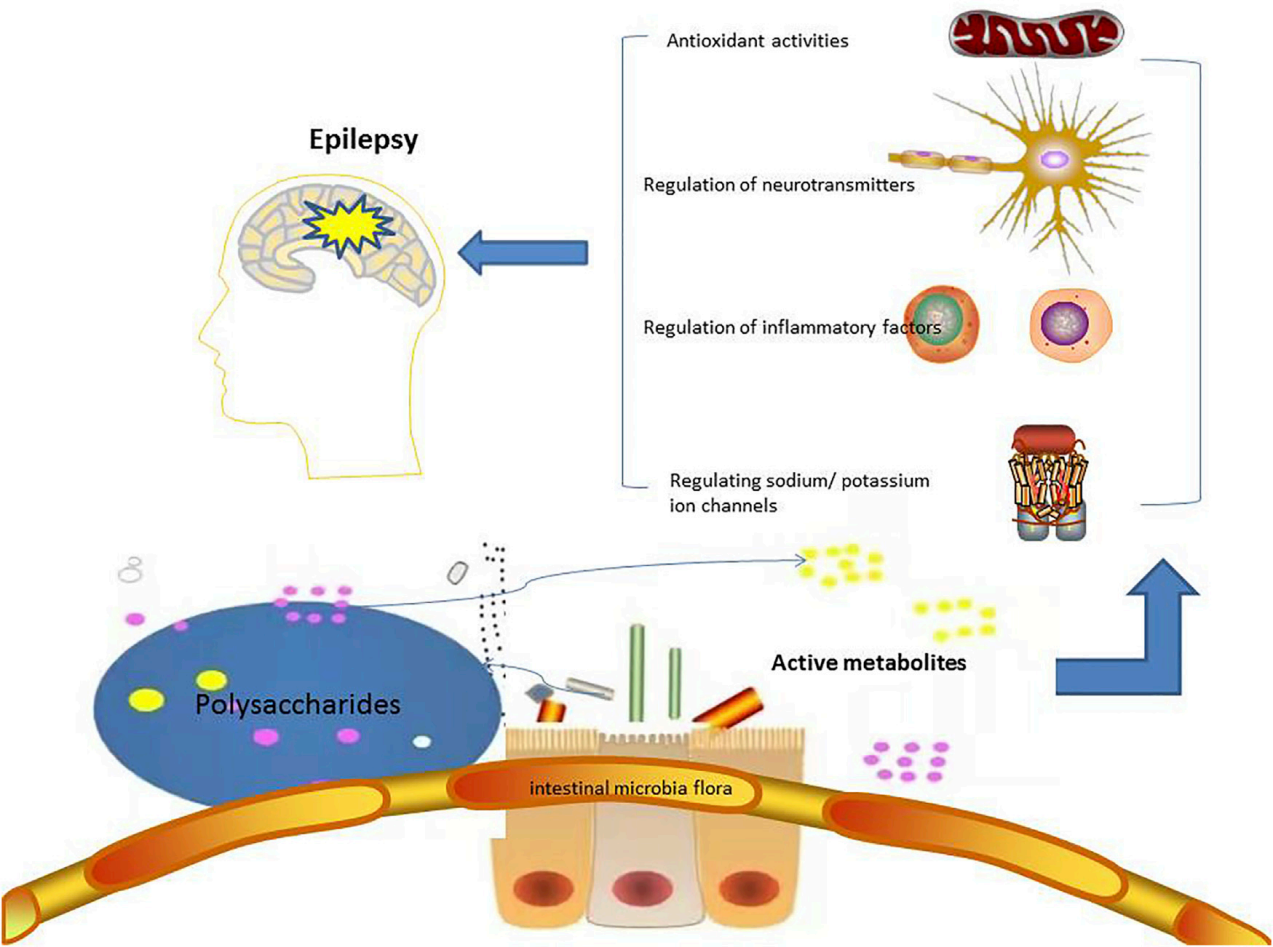
The potential anti-epileptic mechanism of polysaccharides targeting on gut microbiota. Polysaccharides are degraded into active metabolites by gut microbiota, and then to treat epilepsy in the ways of regulating inflammatory factors, neurotransmitters, ion channels, and antioxidant activites.

The relationship among polysaccharides, intestinal flora and human health have been well reviewed and summarized (Liu et al., 2019; Song et al., 2021; Liang et al., 2021; Ma et al., 2021; Yin et al., 2020; Su et al., 2021). Polysaccharides could improve intestinal microecology by repairing intestinal barrier function, regulating the composition of intestinal flora, and regulating intestinal cytokine levels. Under normal circumstances, polysaccharides from TCMs can increase the number of beneficial bacteria such as *Bacteroides*, Firmicutes, and lactic acid bacteria, and reduce the number of harmful bacteria such as *Enterococcus* and *Fusobacterium*. It has also been detected that Chinese medicine polysaccharides can affect TNF-α, slgA, and NF⁃κB and other disease-related changes in biochemical indicators (Zhou et al., 2019). The effects of gut microbiota on epilepsy and potential anti-epileptic mechanism of polysaccharides were shown in Figure 1.

## 4 Conclusion

Current studies support the hypothesis that polysaccharides could be beneficial to the treatment of epilepsy. The evidence includes: 1) polysaccharides have the abilities to regulate inflammatory factors, neurotransmitters, ion channels, enhance immune function, promote the growth of intestinal flora and antioxidant responses. 2) polysaccharide can improve the gastrointestinal health function of the body, regulate the composition of intestinal flora, reshape the intestinal flora ecology and finally to produce anti-epileptic effects. Studies have shown that microbiota intervention could control seizures in animal models. However, in patients with epilepsy, polysaccharides as the next treatment of epilepsy drugs need to be comprehensively investigated with or without combination of anti-epilepsy drugs.

## Data Availability

The original contributions presented in the study are included in the article/Supplementary Material, further inquiries can be directed to the corresponding authors.
